# Extraction and Separation of Eight Ginsenosides from Flower Buds of *Panax Ginseng* Using Aqueous Ionic Liquid-Based Ultrasonic-Assisted Extraction Coupled with an Aqueous Biphasic System

**DOI:** 10.3390/molecules24040778

**Published:** 2019-02-21

**Authors:** Qing Liang, Jinsong Zhang, Xingguang Su, Qingwei Meng, Jianpeng Dou

**Affiliations:** 1 College of Life Sciences, Jilin University, Changchun 130012, China; liangqing@jlu.edu.cn; 2 College of Biological and Agricultural Engineering, Jilin University, Changchun 130022, China; jluzjs@jlu.edu.cn; 3 College of Chemistry, Jilin University, Changchun 130012, China; suxg@jlu.edu.cn; 4 Plusone Native Co., Ltd., Changchun 130012, China; mengqingwei@plusone-intl.com

**Keywords:** response surface methodology, ionic liquids, ginsenosides, ginseng flower buds, ultrasonic-assisted extraction, aqueous biphasic system, separation

## Abstract

Ionic liquids (ILs) are recognized as a possible replacement of traditional organic solvents, and ILs have been widely applied to extract various compounds. The present work aims to extract ginsenosides from *Panax ginseng* flower buds using aqueous ionic liquid based ultrasonic assisted extraction (IL-UAE). The extraction yields of 1-alkyl-3-methylimidazolium ionic liquids with different anions and alkyl chains were evaluated. The extraction parameters of eight ginsenosides were optimized by utilizing response surface methodology (RSM). The model demonstrated that a high yield of total ginsenosides could be obtained using IL-UAE, and the optimum extraction parameters were 0.23 M [C_4_mim][BF_4_], ultrasonic time of 23 min, temperature of extraction set to 30 °C, and liquid-solid ratio of 31:1. After that, an aqueous biphasic system (ABS) was used to separate ginsenosides further. The nature and concentration of salt, as well as the value of pH in ionic liquid were evaluated, and the optimal conditions (6.0 mL IL extract, 3 g NaH_2_PO_4_, and pH 5.0) were obtained. The preconcentration factor was 2.58, and extraction efficiency reached 64.53%. The results indicate that as a simple and efficient method, an IL-UAE-ABS can be considered as a promising method for extracting and separating the natural active compounds from medicinal herbs.

## 1. Introduction

Ginseng has been used in Chinese herbology for thousands of years [1]. Ginseng has many bioactive ingredients, including various ginsenosides. It has been demonstrated that ginseng can prevent and cure a variety of human illnesses, strengthen the body’s resistance, and treat fatigue and diabetes [2,3,4]. Tea made from the flower buds of *Panax ginseng* has become popular as an anti-aging nutritive for older people [5]. The ginsenosides, which are major active constituents in ginseng flower buds, were proven to have good pharmacological effect in lowering blood pressure and anti-inflammation [6,7,8]. Previous phytochemical studies showed that the dammarane-type triterpene saponins such as ginsenoside -Rg1, -Rg2, -Rc, -Rd, -Re, -Rf, -Rb1, and -Rb2, which were often applied to the quality control of ginseng flower buds, were mainly responsible for the wide biological activity [9,10].

In order to study or take full advantage of a natural medicinal plant, extraction and separation techniques are often indispensable. Heat reflux extraction and Soxhlet extraction are common conventional extraction methods, but there are many drawbacks such as time taken and high volumes of organic solvent. Over the years, a requirement for extraction and separation methods that are less time-consuming, require less organic solvent, and with higher extraction efficiency have been sought.

Ionic liquids (ILs) are salts in liquid state, and these molten salts’ melting points are below 100 °C. Organic cations as well as inorganic anions are usual components of ILs. Differing from traditional volatile organic solvents and water, there are distinctive characteristics of ILs, such as negligible volatility, low flammability, high thermal and high ionic conductivity, high polarity, and chemical stability [11,12,13]. ILs have served as green solvents to replace volatile organic solvents in many fields [14]. Additionally, there are some assisted technologies coupled with ILs, like microwave-assisted extraction (MAE) [15], ultrasonic-assisted extraction (UAE) [16], ultrahigh pressure extraction (UPE) [17], as well as negative pressure cavitation-assisted extraction (NPCE) [18], showing notable improvements in the fields of extraction and separation. Ionic liquid based ultrasonic assisted extraction (IL-UAE) has been proven as a more effective method in extracting various compounds from natural plants, such as alkaloids [19], piperine [20], and glabridin [21]. IL-UAE efficiency may be affected by numerous factors, including temperature, liquid-solid ratio, as well as IL concentration. Although the expected response could be affected by many factors and interactions, the response surface methodology (RSM) is proven to be an efficient way to find optimum conditions [22]. RSM has become a prevalent approach to optimize solid-liquid extraction [23,24].

Rogers proposed an aqueous biphasic system based on ionic liquids (IL-ABS) [25], which has been improved to apply in the field of extraction as well as separation for some active compounds, like flavonoids [26], alkaloids [27], and gallic acid [28]. In particular, the IL-ABS could be used to further separate and purify target compounds after IL has been used to extract natural components.

A fast and efficient IL-based UAE extraction method to extract ginsenosides from flower buds and an IL-ABS method used for the preconcentration and separation of ginsenosides were developed in this paper. Firstly, using the IL-UAE method, we extracted ginsenosides from ginseng flower buds with IL aqueous solution, then, ginsenosides were further separated using the IL-ABS. The optimal extraction yields were obtained by using RSM optimizing the factors which had effects on IL-UAE. To improve the efficiency of extraction to the maximum, the parameters in the operation of the IL-ABS were also studied.

## 2. Results and Discussion

### 2.1. Selection of IL

**Figure 1:**
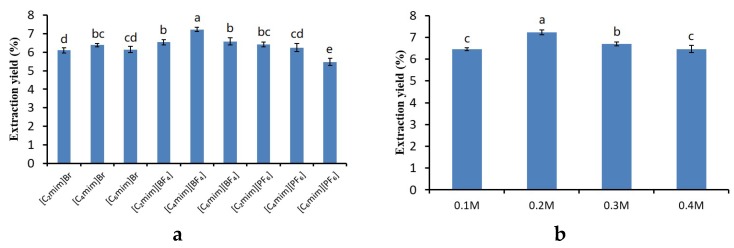
(**a**) Effects of ionic liquids (ILs) with different anions and cations on extraction yields of total ginsenosides; (**b**) Effects of [C_4_mim][BF_4_] with different concentrations on extraction yields of total ginsenosides. Different letters in the same series indicate significant difference at *p* < 0.05.

IL is a type of solvent applied in the extraction and purification fields which is considered environmentally safe. The structure of IL can affect its physicochemical properties remarkably. The structure might also have a great effect on the efficiency when extracting the target compounds [29]. The effect of nine kinds of 1-alkyl-3-methylimidazolium ionic liquids with different anions or cations on the extraction yields of total ginsenosides from flower buds of *Panax Ginseng* were studied in this work. Other conditions include a liquid-solid ratio of 10:1 and executing the extraction at 30 °C for 20 minutes. There was evidence which indicated that the elements affecting the capability of extraction were extremely complex, such as hydrogen-bonding ability, which could become a key factor when the anions are considered [30]; π-π as well as n-π interactions also needed to be noticed when taking imidazole ring containing cations into consideration [31]. The anion of ionic liquid was considered to be the most dramatic factor and had an important effect on their properties. The 1-alkyl-3-methylimidazolium ionic liquids with different anions (Br^−^, [BF_4_]^−^ and [PF_6_]^−^), cations ([C_n_mim], (*n* = 2, 4, and 6)) were studied, and the concentration of different ILs in water was 0.2 M. The extraction yields of ILs with different anions and cations are given in Figure 1a. The results showed that both hydrophilic (Br^−^ and [BF_4_]^−^) and hydrophobic ionic liquids ([PF_6_]^−^) could be used to extract ginsenosides. This is due to the fact that the ginsenoside is composed of hydrophobic sapogenin and hydrophilic glycoside. Although both Br^-^ and [BF_4_]^−^ contained hydrophilic, the ability of [BF_4_]^−^ to form hydrogen-bonding with water molecules was weaker than Br^−^, which contributes to the effective permeation of solution into the plant cells for the dissolution of active components. IL with [BF_4_]^−^ had a prominent extraction yield of total ginsenosides among these ILs. A series of 1-alkyl-3-methylimidazolium cations ([C_n_mim], (*n* = 2, 4, and 6) with the same anion of [BF_4_]^−^ were investigated. The alkyl chain’s length was related to the water miscibility of the [BF_4_]^−^, which might influence the extraction yield [32]. Figure 1a shows that the amount of the extracted total ginsenosides increased significantly with an increase of the alkyl chain length from C2 to C4 but declined with a further increase of alkyl chain length from C4 to C6. The results indicated that C4 showed better solubility of ginsenosides than C2, and steric effects and hydrophobicity increased with an increase of carbon chain length from C4 to C6. Considering the effects of both anion and cation, [C_4_mim][BF_4_] was chosen for the later experiments. In addition, [C_4_mim][BF_4_] was also frequently applied as the extractant in IL-UAE [33,34]. Four different concentrations of [C_4_mim][BF_4_] were studied for extracting total ginsenosides from flower buds of *Panax Ginseng*. The extraction yield of total ginsenosides increased significantly when the concentration of [C_4_mim][BF_4_] was increased from 0.1 and 0.2 M, but declined with further increasing [C_4_mim][BF_4_] concentration from 0.2 to 0.4 M. Consequently, 0.2 M of [C_4_mim][BF_4_] showing better extraction yield was selected for further study.

### 2.2. Optimization of IL-UAE

#### 2.2.1. Single Factor Analysis

**Figure 2:**
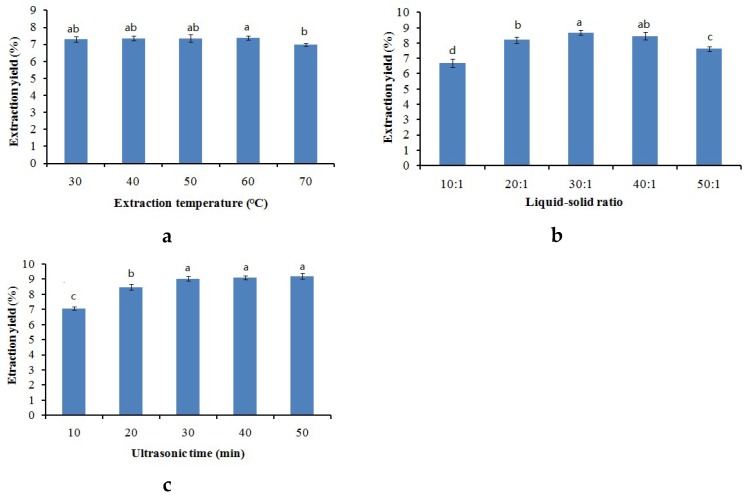
Extraction yields of ionic liquid based ultrasonic assisted extraction (IL-UAE) affected by (**a**) extraction temperature; (**b**) liquid-solid ratio; and (**c**) ultrasonic time. Different letters in the same series indicate significant difference at *p* < 0.05.

Previous studies showed that concentration of solvent, temperature, liquid-solid ratio, as well as extraction duration were the main parameters affecting the extraction yield [21,35]. Thus, these conditions were optimized so that the maximal ginsenosides extraction yield could be achieved. Extraction temperature ranging from 30 to 70 °C with 0.2 M [C_4_mim][BF_4_] was investigated to make its effect clear. Other conditions were liquid-solid ratio set to 10:1 and extracted for 20 minutes. As shown in Figure 2a, compared with the extraction yield from 30 to 60 °C, it was lower at 70 °C, and the extraction yields varied little from 30 to 60 °C. The result may be caused by degradation processes during high temperature which leads to lower extraction yield. Therefore, the IL-UAE was performed at 30 °C.

Liquid-solid ratio was also a crucial factor influencing the extraction efficiency, because when liquid-solid ratio is set too high, it might consume excessive ILs, and a lower ratio may only extract ginsenosides partially. In order to get the optimal liquid-solid ratio, five different liquid-solid ratios of 10:1~50:1 with 0.2 M [C_4_mim][BF_4_] were used to help determine how liquid-solid ratio could influence the extraction yield. Other conditions were ultrasonic time of 20 min and extracting at 30 °C. The result in Figure 2b shows that the extraction yield reached maximum value at the liquid-solid ratio of 30:1. The reason for this might be that with the volume of [C_4_mim][BF_4_] increasing, the material swells more due to absorption of water and effective constituent [36]. Thus, this value (30:1) was chosen for further study.

Generally speaking, the longer an extraction is performed, the higher the extraction efficiency is. Five different extraction times including 10, 20, 30, 40, and 50 minutes were researched with the same extraction parameters: concentration of 0.2 M [C_4_mim][BF_4_], extracting at the temperature of 30 °C, and liquid-solid ratio of 30:1. Figure 2c shows the results from high-performance liquid chromatography (HPLC) analysis, which demonstrates that the extraction yields of the eight ginsenosides increased with the increase of ultrasonic time, but that there was no obvious difference among the extraction yields of total saponins (*p* > 0.05) when the UAE executing time increased from 30 to 50 minutes. The diffusion front moving towards the interior of the tissues caused a decrease of diffusion area and an increase of diffusion distance, which explains the reduction of diffusion rate [37]. Ultimately, the most appropriate ultrasonic time was set as 30 min.

This single factor IL-UAE experiment indicated that using [C_4_mim][BF_4_] as the extraction solvent, concentration at 0.2 M, extracting at the temperature of 30 °C, liquid-solid ratio of 30:1, and 30 minutes’ ultrasonic treatment were the optimal conditions for extraction of eight ginsenosides.

#### 2.2.2. Experimental Design for Optimization and Statistical Analysis

Because the extraction process is usually affected by multiple parameters, a key preliminary procedure is to select and optimize the experimental conditions in the development of IL-UAE methods. In this study, the optimum conditions for IL-UAE of ginsenosides were determined by using RSM. The optimal experimental conditions were determined using the Design-Expert (DE) 8.0 software (Statease Inc., Minneapolis, MN, USA). A Box-Behnken design (BBD) with three independent variables was applied [38]. The variables used were: liquid-solid ratio (mL/g, A), ultrasonic time (min, B), and IL concentration (M, C); each variable was set at three levels. The extraction temperature selected was 30 °C. Table 1 exhibits the different experimental conditions and the corresponding results of extraction yields.

To control and minimize the impact of unaccountable variability in the observed reactions, every experiment was executed randomly. Multiple regressions were used to analyze laboratory data so as to fit the second order regression equation. An independent variable equation can be obtained by using marked factors to form the regression model:

Y = 9.36 − 0.12A − 0.26B + 0.66C + 0.11AB + 0.23AC − 0.13BC− 1.25A^2^ − 0.31B^2^ − 1.01C^2^ (1)

where Y is the dependent variable of extraction yield; A, B, and C are the independent variables (liquid-solid ratio, IL concentration, and ultrasonic time).

The results of fitting models to the data are presented in Table 2. The correlation coefficient value was used to estimate the quality of the model proposed in the paper. The coefficient of determination (R^2^) was 0.9942, which was obtained from the calculated model, showing that the model can describe the real relation among the selected parameters nearly precisely. F-value and *p*-value are used to indicate the significance of coefficients. It is suggested that as the F-value increases and *p*-value decreases, the effect on the corresponding response variable is more obvious [39]. The variables which have the most significant influences on extraction yield were the linear term of IL concentration (C), and the quadratic terms of liquid-solid ratio (A^2^) and IL concentration (C^2^). The liquid-solid ratio (A) and time (B) are linear terms; time (B^2^), the interaction term of liquid-solid ratio and IL concentration (AC), which are the quadratic terms, were also apparent at 5% level. Conversely, the interaction between liquid-solid ratio and time (AB) and time and IL concentration (BC) did not show evident effects on the extraction yield of IL-UAE of ginsenosides (*p* > 0.05). It is suggested the model was efficient with an F-value equaling 95.77 and a *p*-value smaller than 0.0001, and the possibility that the “Model F-value” reached this level because of noise was only 0.01%. The value of “Lack of Fit” was not significant when *p* was greater than 0.05. The significance of the model terms was indicated when “Prob > F” was lower than 0.05. The consequences above indicated that the developed model was effective when used for predicting the response.

**Figure 3:**
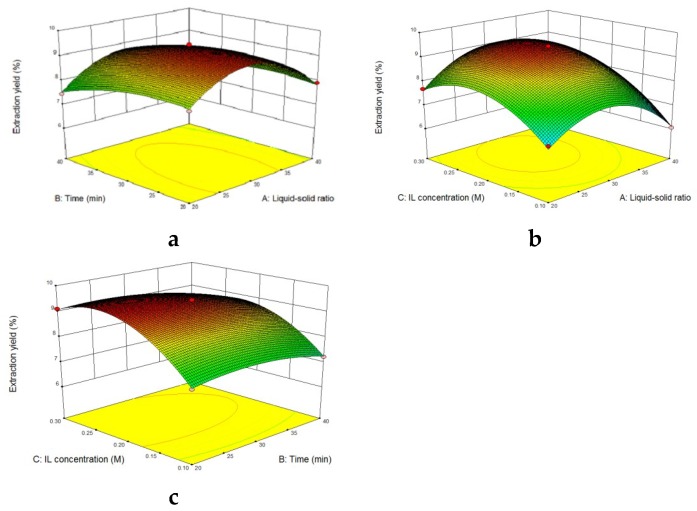
Response surface and contour plots presenting effects of liquid-solid ratio, time, and IL concentration on extraction yields of total ginsenosides using IL-UAE. (**a**) liquid-solid ratio and time; (**b**) liquid-solid ratio and IL concentration; (**c**) time and IL concentration.

Figure 3 illustrates the relationship between independent and dependent variables using 3D representation of the response surfaces. These plots depicted two variables within experimental range when the third variable kept constant at zero levels. The experiments were repeated three times in the practical scenarios using the values for the optimum conditions that the DE software suggested (31:1 liquid-solid ratio; 23 min ultrasonic time, and 0.23 M IL concentration) in regular applications. The experimental data (9.58%) was fully approached to the predicted value (9.43%). The verification experiments have confirmed that the proposed model could well reflect the desired optimization.

### 2.3. Comparison of Different UAE Methods

IL-UAE was compared with UAE using water (Water-UAE), and UAE using methanol (Methanol-UAE) to assess its extraction efficiency of ginsenosides. The concentration of [C_4_mim][BF_4_] was 0.2 M, and the concentration of methanol was 60%. The liquid-solid ratios used to extract were 30:1 uniformly. Other conditions were as follows: extraction temperature set to 30 °C and ultrasonic time of 30 min. The extraction yields of the three methods were 9.12% (IL-UAE), 4.31% (Water-UAE), and 8.69% (Methanol-UAE). The results showed the IL-UAE method reached the highest extraction yield of total ginsenosides among these three methods, which meant that IL-UAE is an efficient and speedy technique in preparing samples for extraction of ginsenosides from ginseng flower buds. As a result, the substitution of conventional volatile organic solvents using IL is practicable.

### 2.4. Preconcentration and Separation of Eight Ginsenosides Using IL-ABS

**Figure 4:**
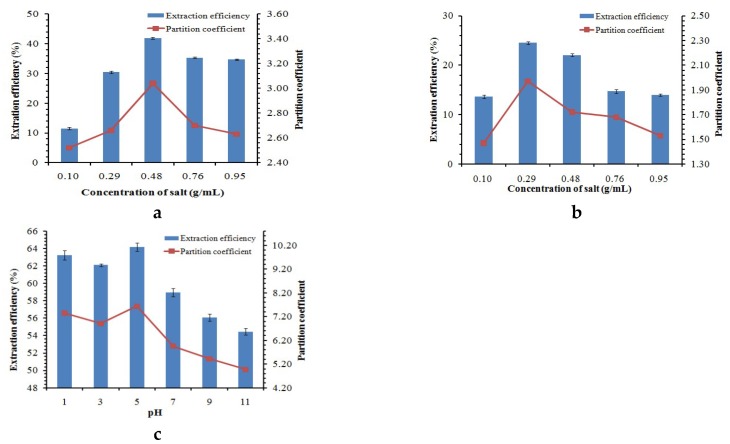
Effect of (**a**) NaH_2_PO_4__,_ (**b**) (NH_4_)_2_SO_4,_ (**c**) system pH at room temperature on the extraction efficiencies and partition coefficient.

The further process of isolating and purifying ginsenosides was conducted using an aqueous biphasic system (ABS), and the ABS was realized by adding various salts to the IL extraction. The dominant factors affecting the IL-ABS extraction were researched to obtain the optimal conditions, including the type and concentration of salt and pH. The salts NaH_2_PO_4_ and (NH_4_)_2_SO_4_ were selected as elements to form an ABS. Figure 4a,b show the partition coefficients and extraction efficiency of ABSs made up by the salts. The results indicate that the extraction efficiencies and the partition coefficients initially increased to a maximum with salt concentration increase, but later decreased as the salt concentration continued to increase. The maximum of the extraction efficiencies of NaH_2_PO_4_ and (NH_4_)_2_SO_4_ were 41.98% and 24.53% for IL-ABS extraction, respectively. The maximum of the partition coefficients of NaH_2_PO_4_ and (NH_4_)_2_SO_4_ were 3.04 and 1.97, respectively. The extraction efficiency and the partition coefficient of NaH_2_PO_4_ were higher than (NH_4_)_2_SO_4_ for ginsenosides under the optimum conditions.

To determine the influence of pH, values of pH varying from 1.0 to 11.0 were tested for [C_4_mim][BF_4_]/NaH_2_PO_4_ ABS. Figure 4c demonstrates that the partition coefficient and the extraction efficiency achieved a maximum at pH 5.0 and declined as the alkalinity of the solution increased. The optimizing extraction efficiency was attained at a maximum of 64.53% under acidic conditions at pH 5.0, and the partition efficient was 7.64. The result indicated that pH was an important factor for the preconcentration of ginsenosides using the IL-ABS. The optimum conditions were 6.0 mL IL extract of ginseng flower buds, 3.0 g NaH_2_PO_4_, and pH 5.0. Under the optimized conditions, the concentration of total ginsenosides in IL-phase increased, and the preconcentration factor for eight ginsenosides reached 2.58.

## 3. Materials and Methods

### 3.1. Materials and Reagents

All ionic liquids employed in the test, including [C_n_mim]Br (n = 2, 4, and 6, 1-alkyl-3-methylimidazolium bromide), [C_n_mim] [BF_4_] (n = 2, 4, and 6, 1-alkyl-3-methylimidazolium tetrafluoroborate), [C_n_mim] [PF_6_] (n = 2, 4, and 6, 1-alkyl-3-methylimidazolium hexafluorophosphate), were purchased from Lanzhou Zhongke Kaite Science, Industry & Trade Co. Ltd., Lanzhou, China. A microporous membrane (φ 13 mm, 0.22 μm) produced by Tianjin Tengda Filtration Instrument Co. Ltd. (Tianjin, China) was chosen. Ginsenosides (Rg_1_, Re, Rf, Rg_2_, Rb_1_, Rc, Rb_2_, Rd) were purchased from the National Institute for the Control of Pharmaceutical and Biological Products. Standard compound purity was higher than 98%. Acetonitrile (HPLC grade) was produced by Thermo Fisher Scientific, Inc. (Waltham, MA, USA). Phosphoric acid (99.7%) was bought from TEDIA (Fairfield, OH, USA). An ultra clear water purification system (Merck Millpore, Waltham, MA, USA) was used to purify the water. The flower buds of *Panax ginseng* were bought from Jilin Plusone Native Co., Ltd. (Changchun, China). A disintegrator was used to crush the ginseng flower buds into powder, and then the powder was sifted using 80 mesh stainless steel sieves. The air drier temperature for the powder samples was 60 °C, then the samples were preserved in a dry and dark environment until used. Other chemicals used were analytical reagent-grade quality.

### 3.2. Sample Preparation

UAE of ginsenosides was carried out using IL aqueous solution. A given liquid-solid ratio was reached by adding a specific amount of IL aqueous solution and ginseng flower buds powder into a tube, then the tube was put in a KQ-250DE ultrasonic instrument (Kunshan Ultrasound Co. Ltd., Jiangsu, China) to extract the ginsenosides. The electric power was set at 250 W and the generator’s frequency was 40 kHz. The conditions that IL-UAE was executed at were anion: Br, BF_4_, and PF_6_; cation: C_2_, C_4_, and C_6_; IL concentration (M): 0.1, 0.2, 0.3, and 0.4; solvent: water, methanol and ILs; temperature (°C): 30, 40, 50, 60, and 70; liquid-solid ratio (mL/g): 10:1, 20:1, 30:1, 40:1, and 50:1; extraction time (minutes): 10, 20, 30, 40, and 50. The sediment was then removed by using a centrifuge machine set at 4,000 × g for 5 minutes. Further analysis of the content of the eight saponins used the supernatant. All samples were prepared in triplicate to be analyzed in the experiments. The extraction yield (E) of total ginsenosides was calculated according to Equation (2):

(2) $$E=\frac{m}{{\;m}_{0}}\times 100\%,$$

where m is the mass of total ginsenosides in the extraction solution and m_0_ is the mass of the sample.

### 3.3. HPLC/UV Analysis

**Figure 5:**
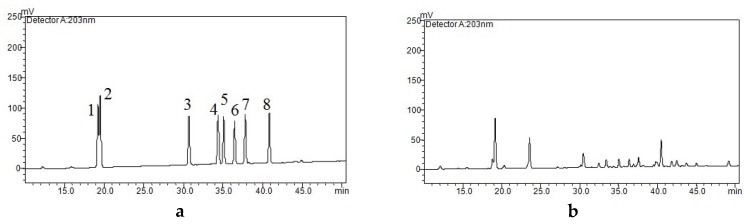
HPLC profiles of (**a**) the standard mixture solution and (**b**) a sample prepared using IL-UAE from ginseng flower buds. Peaks 1–8 correspond to ginsenoside Rg_1_, Re, Rf, Rg_2_, Rb_1_, Rc, Rb_2_, and Rd, respectively.

HPLC analysis was executed using a Shimadzu LC-20A liquid chromatographic system (Shimadzu Technologies, Japan). A 150 × 4.6 mm i.d., 5 μm, ODS C18 column from Shimadzu Technologies was employed to perform the separation. The mobile phase consisted of (A) water containing 0.5% phosphoric acid and (B) acetonitrile. The program for gradient elution was: 0–20 min, 20–22% B; 20–23 min, 22–28% B; 23–45 min, 28–35% B; 45–55 min, 35% B. For all the experiments, the column temperature was set at 35 °C, the flow rate was 1.0 mL/min, and the injection volume was 10 μL. The UV absorbance detection wavelength was 203 nm, and all samples were filtered using a 0.22 µm syringe membrane filter before HPLC analysis. Identification of eight ginsenosides was on the basis of retention time compared with standards. By comparing the absorbance recorded in the chromatograms with the external standards, the quantification of ginsenosides was realized. The concentration of each ginsenoside in the samples was computed from peak area on the basis of calibration curves. Figure 5 shows the HPLC chromatograms of the standard mixture solution and a sample using IL-UAE.

### 3.4. Aqueous Biphasic System (ABS)

A 6 mL IL extract (which included 0.23 M IL), 0.6 g IL (in order to gain a higher enrichment factor), and a suitable amount of salt were added to a 15 mL graduated tube. When the effect of pH was studied, Britton‒Robinson (B‒R) buffer solution was used to adjust pH values. The mixture was vortexed and centrifuged at 3000× *g* for 2 min after completing dissolution of salt. The mixture stood for 10 min, and the two-phase volumes could be read. IL-rich phase and salt-rich phase were the two distinct phases observed with the volume of each being marked. The content of eight ginsenosides in the IL phase were calculated individually, and mass balance was applied in the calculation of the content in the salt solution phase. The extraction efficiency (EE) of total ginsenosides of the IL phase was calculated using the following Equation (3):

(3) $$EE=\frac{m_{\mathrm{IL}}}{m_{1}}\times 100\%,$$

where m_IL_ and m_1_ are the mass of total ginsenosides in the IL phase before and after ABS extraction, respectively. The partition coefficient (K) derives from the following Equation (4):

(4) $$K=\frac{C_{\mathrm{IL}}}{C_{s}},$$

where C_IL_ is total ginsenosides content in the IL phase, and C_s_ is total ginsenosides content in the salt solution phase. The preconcentration factor (PF) of total ginsenosides is defined according to Equation (5):

(5) $$PF=\frac{C_{\mathrm{IL}}}{C_{0}},$$

where C_IL_ represents total content of ginsenosides before ABS extraction, and C_0_ is the total content of ginsenosides after the extraction in the IL phase.

### 3.5. Statistical Analysis

Design Expert 8.0 (DE, Statease Inc., Minneapolis, MN, USA) was used to analyze the experimental data and obtain the response models. The optimized conditions for the variables were acquired using statistical analysis. The statistical analysis for screening the ILs and single factor experiments was done using Duncan’s multiple range test. Each error bar indicates the standard deviation of triplicate tests.

## 4. Conclusions

IL-UAE coupled with an ABS was used to extract eight ginsenosides from the flower buds of *Panax ginseng* in this paper. The results of the experiments illustrated that IL was a superb extractant for the UAE process, and the best IL for extracting ginsenosides from ginseng flower buds was [C_4_mim][BF_4_]. RSM was applied to optimize extraction conditions, including IL concentration, liquid-solid ratio, and ultrasonic time. Under these optimal conditions (0.23 M [C_4_mim][BF_4_], temperature set to 30 °C, liquid-solid ratio of 31:1, and executing extraction for 23 minutes), the method could help to reach a higher extraction yield of total ginsenosides. The presented IL-UAE method is a promising method for preparing samples of medicinal plants in consideration of the physicochemical properties of ILs. Using the ABS for further purification of total ginsenosides, a preconcentration factor of 2.58 was obtained, with a notable extraction efficiency of 64.53% and partition coefficient of 7.64. The experimental data showed that IL-UAE coupled with an ABS could be used not only in extraction but also in further separating pharmaceutical components from various natural plant sources.

## Figures and Tables

**Table 1:** Box-Behnken design with the observed response for extraction yield (%) of total ginsenosides using IL-UAE.

Run	A: Liquid-Solid Ratio	B: Time (min)	C: Concentration of IL (M)	Extraction Yield (%)
1	−1 (20:1)	−1 (20)	0 (0.2)	8.19
2	1 (40:1)	−1 (20)	0 (0.2)	7.91
3	−1 (20:1)	1 (40)	0 (0.2)	7.45
4	1 (40:1)	1 (40)	0 (0.2)	7.60
5	−1 (20:1)	0 (30)	−1 (0.1)	6.89
6	1 (40:1)	0 (30)	−1 (0.1)	6.04
7	−1 (20:1)	0 (30)	1 (0.3)	7.69
8	1 (40:1)	0 (30)	1 (0.3)	7.75
9	0 (30:1)	−1 (20)	−1 (0.1)	7.46
10	0 (30:1)	1 (40)	−1 (0.1)	7.22
11	0 (30:1)	−1 (20)	1 (0.3)	9.11
12	0 (30:1)	1 (40)	1 (0.3)	8.34
13	0 (30:1)	0 (30)	0 (0.2)	9.45
14	0 (30:1)	0 (30)	0 (0.2)	9.34
15	0 (30:1)	0 (30)	0 (0.2)	9.28

**Table 2:** Analysis of variance for the response surface of extraction yield (%) of ginsenosides using IL-UAE.

Source	Sum of Squares	DF	Mean Square	F-Value
Model	13.44	9	1.49	95.77 ***
A	0.11	1	0.11	6.78 *
B	0.53	1	0.53	34.02 **
C	3.48	1	3.48	223.48 ***
AB	0.046	1	0.05	2.96 ^NS^
AC	0.21	1	0.21	13.28 *
BC	0.070	1	0.07	4.50 ^NS^
A^2^	5.81	1	5.81	372.70 ***
B^2^	0.37	1	0.37	23.43 **
C^2^	3.76	1	3.76	241.35 ***
Residual	0.078	5	0.02	
Lack of fit	0.063	3	0.02	2.83^NS^
Pure Error	0.015	2	7.43 × 10^3^	
Total	13.52	14		

DF—Degree of Freedom, * *p* < 0.05, ** *p* < 0.01, *** *p* < 0.001, NS—Not Significant.
